# Molecular epidemiology and genetic evolution of avian influenza H5N1 subtype in Nigeria, 2006 to 2021

**DOI:** 10.1007/s11262-024-02080-9

**Published:** 2024-06-19

**Authors:** Ridwan O. Adesola, Bernard A. Onoja, Andrew M. Adamu, Sheriff T. Agbaje, Modinat D. Abdulazeez, Olalekan C. Akinsulie, Adetolase Bakre, Oyelola A. Adegboye

**Affiliations:** 1 Department of Veterinary Medicine, Faculty of Veterinary Medicine, University of Ibadan, Ibadan, 200005 Nigeria; 2 Department of Virology, Faculty of Basic Medical Sciences, College of Medicine, University of Ibadan, Ibadan, 200005 Nigeria; 3 College of Public Health, Medical and Veterinary Sciences, James Cook University, Townsville, QLD 4811 Australia; 4 Australia Institute of Tropic Health and Medicine, James Cook University, Townsville, QLD 4811 Australia; 5 Department of Veterinary Public Health and Preventive Medicine, University of Abuja, Abuja, 900105 Nigeria; 6 Department of Statistics, Faculty of Science, University of Ibadan, Ibadan, 200005 Nigeria; 7 College of Veterinary Medicine, Washington State University, Pullman, WA USA; 8 Menzies School of Health Research, Charles Darwin University, Darwin, NT 0811 Australia

**Keywords:** Avian influenza, H5N1, Epidemiology, Genomics, Evolution, Nigeria

## Abstract

Nigeria recorded one of the earliest outbreaks of the Highly Pathogenic Avian Influenza (HPAI) virus H5N1 in 2006, which spread to other African countries. In 2023, 18 countries reported outbreaks of H5N1 in poultry, with human cases documented in Egypt, Nigeria, and Djibouti. There is limited information on the molecular epidemiology of HPAI H5N1 in Nigeria. We determined the molecular epidemiology and genetic evolution of the virus from 2006 to 2021. We investigated the trend and geographical distribution across Nigeria. The evolutionary history of 61 full-length genomes was performed from 13 countries worldwide, and compared with sequences obtained from the early outbreaks in Nigeria up to 2021. MEGA 11 was used to determine the phylogenetic relationships of H5N1 strains, which revealed close ancestry between sequences in Nigeria and those from other African countries. Clade classification was performed using the subspecies classification tool for Bacterial and Viral Bioinformatics Research Center (BV-BRC) version 3.35.5. H5N1 Clade 2.2 was observed in 2006, with 2.3.2, 2.3.2.1f clades observed afterwards and 2.3.4.4b in 2021. Our findings underscore the need for genomics surveillance to track antigenic variation and clades switching to monitor the epidemiological of the virus and safeguard human and animal health.

**Impacts**

- Specific variations in the hemagglutinin (HA) and neuraminidase (NA) genes of Avian influenza virus are consistent in different geographical regions.
- H5N1 Clade 2.2 was reported in 2006, with 2.3.2, 2.3.2.1f afterwards and 2.3.4.4b in 2021.
- Nigeria is an epicentre for avian influenza with three major migratory routes for wild birds transversing the country.
- It is plausible that the Avian influenza in Northern Nigeria may be linked to wild bird sanctuaries in the region.

**Supplementary Information:**

The online version contains supplementary material available at 10.1007/s11262-024-02080-9.

## Introduction

Avian influenza (AI), also known as bird flu, is a viral disease that affects both domestic and wild birds, causing severe respiratory, digestive, and occasionally neurological symptoms [1]. Avian influenza causes the disease type A virus [2]. Avian influenza viruses (AIVs) belong to the *Orthomyxoviridae* family and possess a single-stranded RNA genome of eight-gene negative-sense genes (PB2, PB1, PA, HA, NP, NA, M, and NS) [3]. The viruses can be divided into subtypes based on the surface proteins, hemagglutinin (HA) and neuraminidase (NA), nine NA (N1 to N9) and 16 HA (H1 to H16) currently identified in avian populations [4]. AI viruses are divided into two pathotypes based on the haemagglutinin cleavage site (HACS) motif and pathogenicity traits in poultry flocks, including chickens: Low pathogenic avian influenza (LPAI) viruses do not have the polybasic HACS motif (pHACS) of the highly pathogenic avian influenza (HPAI) viruses [5]. Further, the intravenous pathogenic index (IVPI) is used to classify the virus as HPAI if this is more than 1.2, and LPAI if it is lower [6]. The HACS motif predominates despite several factors contributing to H5N1 HPAI pathogenicity [5]. Replication of the LPAI viruses occurs in the gastrointestinal tract, kidney, and the epithelial surfaces of the respiratory system [5]. Reverse genetics removes the pHACS motif, which eliminates the feature of HPAI viruses replicating in multiple tissues and overwhelming infection within the vascular compartment of chickens [5]. AIVs are a serious threat to the poultry industry, causing multiple outbreaks [7].

Genetic lineages of HPAI H5N1 subtypes have evolved and spread from progenitors (A/goose/Guandong/96) in China [8]. In 2005, a widespread outbreak in Qinghai Lake, North West China, led to the death of thousands of ducks [9]. The virus spread westward through Central Asia to Europe, the Middle East, and Africa [10]. There are significant concerns about the future of the poultry industry and the public health risks associated with the expansion of H5N1 in Africa. These concerns pertain to food security and the threats to human health, which can be fatal. Human infections are caused by six subtypes namely: H3 (H3N8), H5 (HPAI H5N1, H5N6, and H5N8), H6, H7, H9 (LPAI H9N2), and H10 [11]. Direct contact with infected chickens or surfaces and items contaminated by their feces or secretions is the primary means that AIV is transmitted from poultry to humans. Another theory states that AIV initially infects pigs, after which it spreads to humans by contact with infected pigs’ secretions, skin, blood, and fur [11].

In Africa, Nigeria was the first to report an outbreak of HPAI H5N1 subtype in chickens in Kaduna State [12]. The outbreak persisted for 21 months and spread to 25 out of the 36 States [13]. Subsequently, it spread to eleven African countries, with infections occurring in humans and animals in Egypt [14]. Eight years after the first outbreak, there was a resurgence of HPAI H5N1 in Nigeria in 2015, with the isolation of reassortant strain of H5N8 from live bird markets (LBMs) in Lagos State and backyard poultry in Kano State [15]. This outbreak led to the culling of more than 3.7 million birds nationwide, with an economic loss of over $7.2 million [16]. It spread to Burkina Faso, Cote d’Ivoire, Ghana, Cameroon, and Niger [6].

In 2021, an outbreak of HPAI H5N1 was reported, and currently, three subtypes (H9N2, H5N8 and H5N6) are co-circulating in LBMs in Nigeria [17]. The control and intervention strategies include vaccination, depopulation, culling infected birds, disinfection, and decontamination of farm equipment [16]. Despite vaccination, sporadic outbreaks occur in Nigeria due to poor biosecurity measures, weak surveillance and limited diagnostic capacity. We determined the molecular epidemiology and genetic evolution of HPAI H5N1 in Nigeria to better understand the current trend of AIV.

## Materials and methods

### Determination of avian influenza incidence in Nigeria

Updated cases of AI were retrieved from the database of the Federal Ministry of Agriculture Department of Veterinary and Pest Control Services following surveys in live bird markets in Nigeria [16]. The data was used to determine the incidence and current status of AI across Nigeria.

### Study design for the molecular analysis

FASTA sequences of AIVs were retrieved from the OpenFlu database from 2006 to 2021 [18, 19]. OpenFlu is an open-source global database for AIV operated by the Swiss Institute of Bioinformatics. The sequences were analysed to know the molecular characteristics of AIVs in Nigeria. The analysis was based on the number of AIV sequences deposited from Nigeria, and the percentage of available AIV subtypes.

### Inclusion and exclusion criteria for selected sequence for phylogenetic analysis

The 289 partial sequences in the phylogenetic analysis were based on their length, avian species representative, year, and country of isolation. Only 11 whole genome and H5N1 subtype sequences were included. Sequences of other avian species (such as chicken, duck, goose, turkey, and guinea fowl), year (from 2006 to 2021), and country (Nigerian, South Korea, Vietnam, Japan, Egypt, Cote d’Ivoire, Sudan, Niger, Burkina Faso, Cameroon, Ghana, United States, and Canada) of isolation were included in the analysis. Duplicated sequences or those not within these criteria were excluded from the analysis.

### Phylogenetic analysis

Based on the Tamura 3-parameter model, the evolutionary history was estimated using the Maximum Likelihood model [20]. Using the Maximum Composite Likelihood technique [21], neighbour-joining [22], and BioNJ algorithms on pairwise distance matrices, the topology with the highest log likelihood value was chosen to create the trees. The robustness of phylogenetic diversity was evaluated using 1000 bootstrap replicates. Branch lengths were calculated as the number of substitutions per site, and the trees were drawn to scale. Sixty-one H5N1 sequences were used for the phylogenetic analysis; Nigeria (n = 11), South Korea (n = 15), Vietnam (n = 9), Japan (n = 5), Egypt (n = 8), Cote d’Ivoire (n = 2), Sudan (n = 1), Niger (n = 2), Burkina Faso (n = 2), Cameroon (n = 2), Ghana (n = 1), USA (n = 2), and Canada (n = 1).

### Determination of H5N1 subspecies clade classification

Clade classification was performed on all the sequence data isolated from Nigeria using the subspecies classification tool for Bacterial and Viral Bioinformatics Research Center (BV-BRC) version 3.35.5.

### Ethical approval

Ethical approval was not required for this study.

## Results

### Incidence and molecular characteristics of AIV in Nigeria

**Fig. 1:**
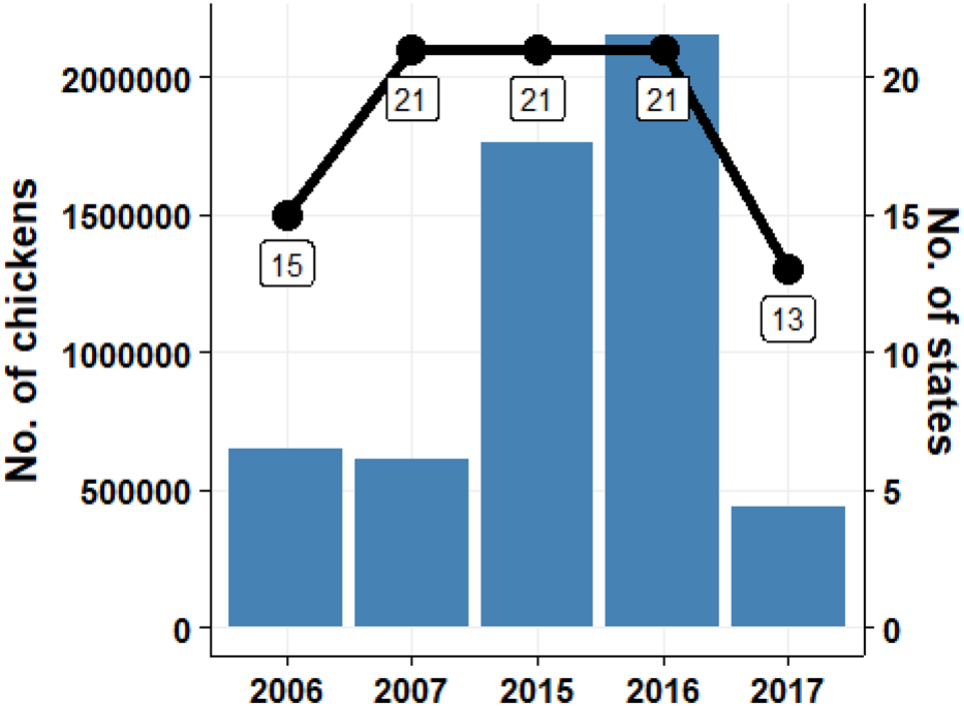
Number of bird deaths (and the number of affected states) between 2006 and 2017

**Fig. 2:**
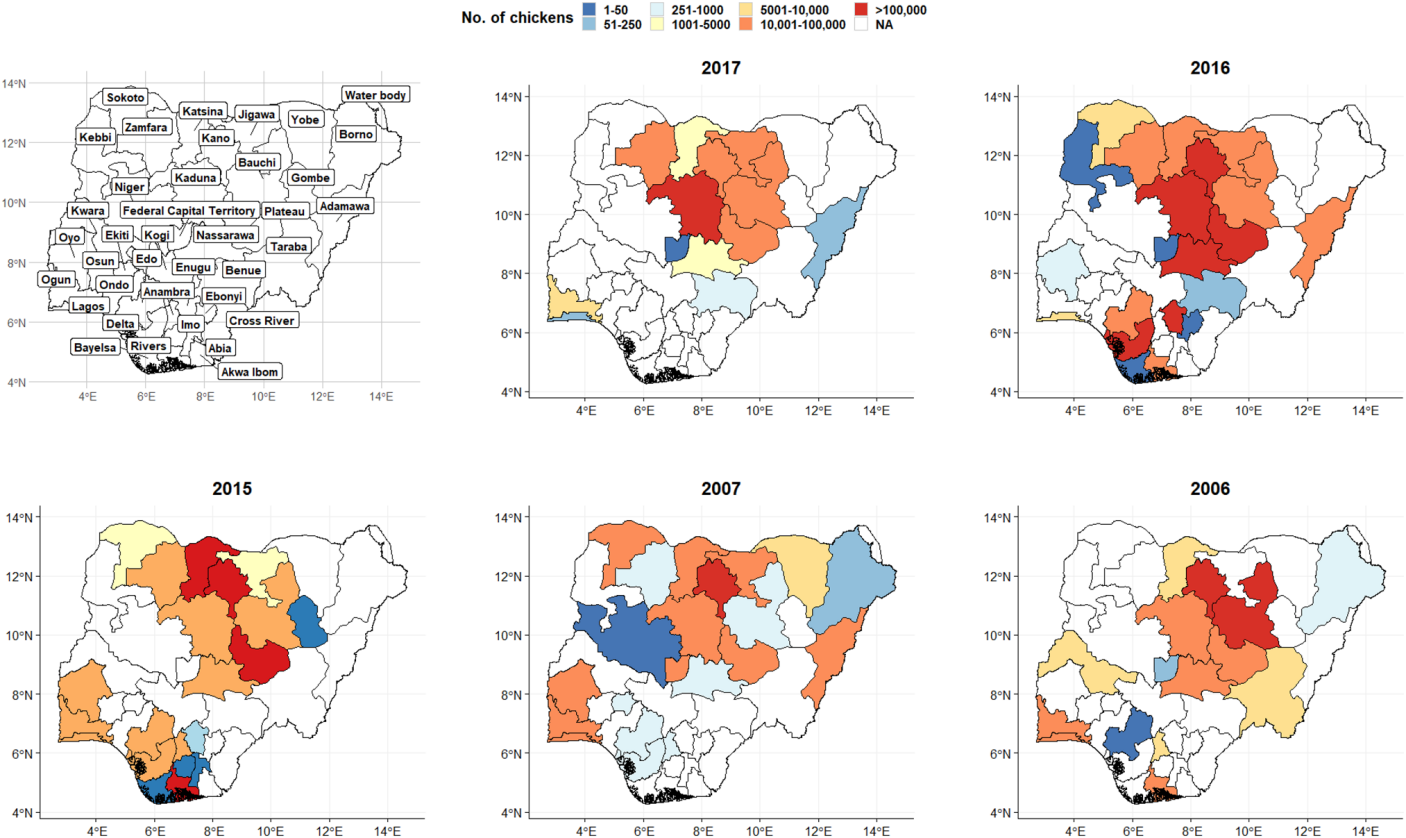
Geographical distribution of AIV birds mortality in Nigeria

From 2006 to 2017, multiple outbreaks of AIV were reported in 32 states of Nigeria, including the Federal capital territory, resulting in the death of over 5.5 million birds (Figs. 1 & 2). Although the disease spread to many states, Kano, Kaduna, and Plateau States were the most affected, accounting for about 60% of all reported cases in Nigeria (Fig. 2). A total of 289 sequences were retrieved from the OpenFlu database (Table S1); H5N1 accounted for 97%, H2H5 (1%), H5N6 (0.3%), and H5N8 (2%).

### Phylogenetic relationship of H5N1

A total of 61 H5N1 complete genomes were obtained from 2006 to 2021, and they were isolated from chicken, duck, goose, turkey, and guinea fowl (Table S2). Specifically, the genomes comprised eleven Nigerian sequences, fifteen South Korean sequences, nine Vietnamese, five Japanese, eight Egyptian, two sequences from Cote d’Ivoire, one sequence from Sudan, two sequences from Niger, two sequences from Burkina Faso, two sequences from Cameroon, one sequence from Ghana, two sequences from the United States, and one sequence from Canada.

**Fig. 3:**
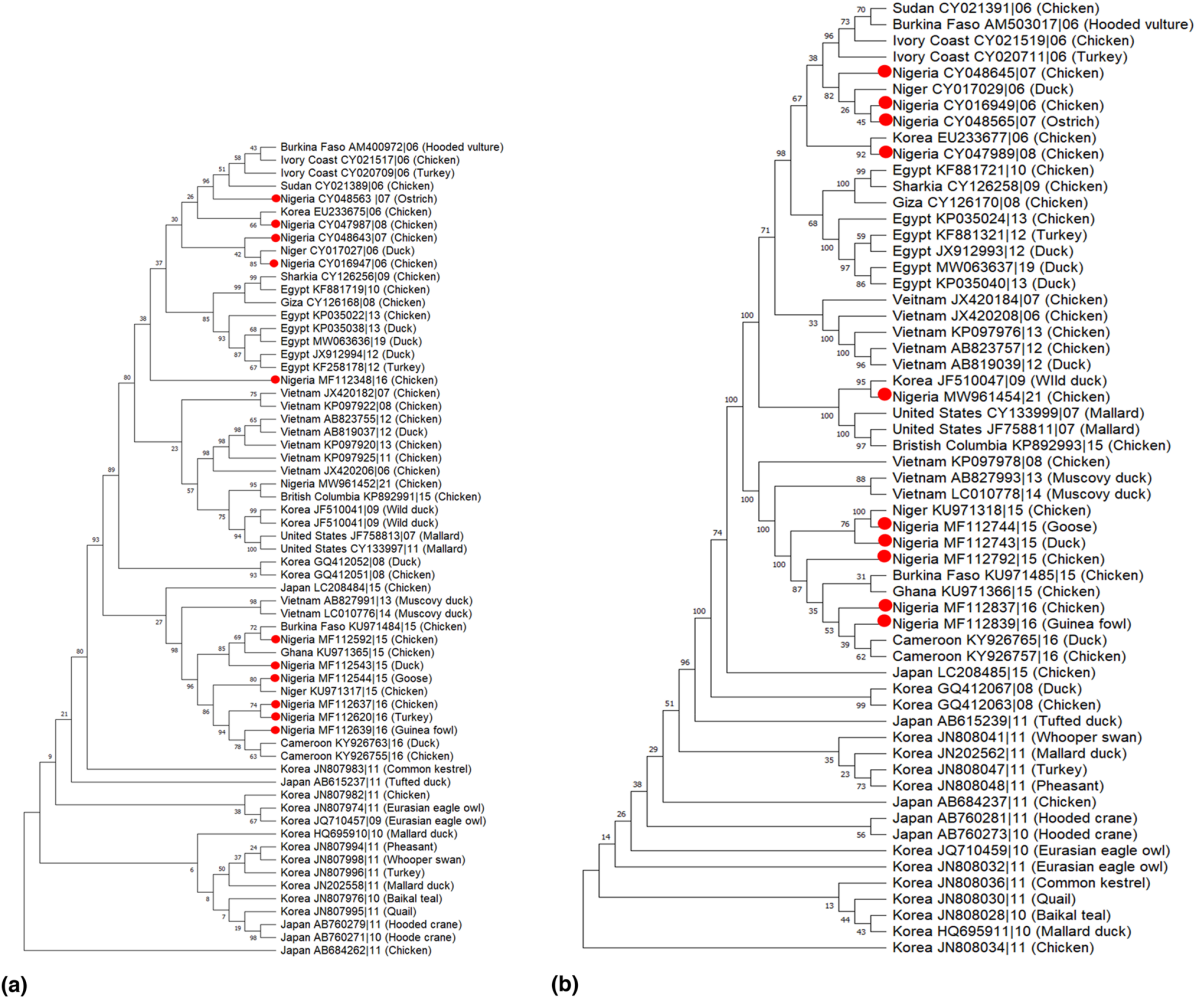
Phylogenetic relationship of H5N1 viruses from Nigeria (red dots) and other countries generated in MEGA11 (maximum likelihood analysis and 1000 bootstrap replicates) for (**a**) HA and (**b**) NA gene

Phylogenetic trees were constructed to show the evolutionary relationship of 8 gene segments (HA, NA, MP, NP, NS, PA, PB1, and PB2) of H5N1 (Fig. 3). The HA sequences from Nigeria clustered with sequences from other African countries (Fig. 3A). Specifically, the AIV detected in Nigeria in 2006, 2007, 2008, and 2016 showed close ancestry with sequences from Burkina Faso (2006), Ivory Coast (2006), Sudan (2006), Niger (2006), Korea (2006), and Egypt (2008, 2009, 2010, 2012, 2013, 2019). These AIVs were isolated from different avian hosts, such as hooded vultures, chickens, turkeys, ostriches, and ducks. A cluster of sequences from Nigeria in 2015 and 2016 were similar to sequences from Ghana in 2015, Burkina Faso in 2015, Niger in 2015, and Cameroon in 2016.

**Fig. 4:**
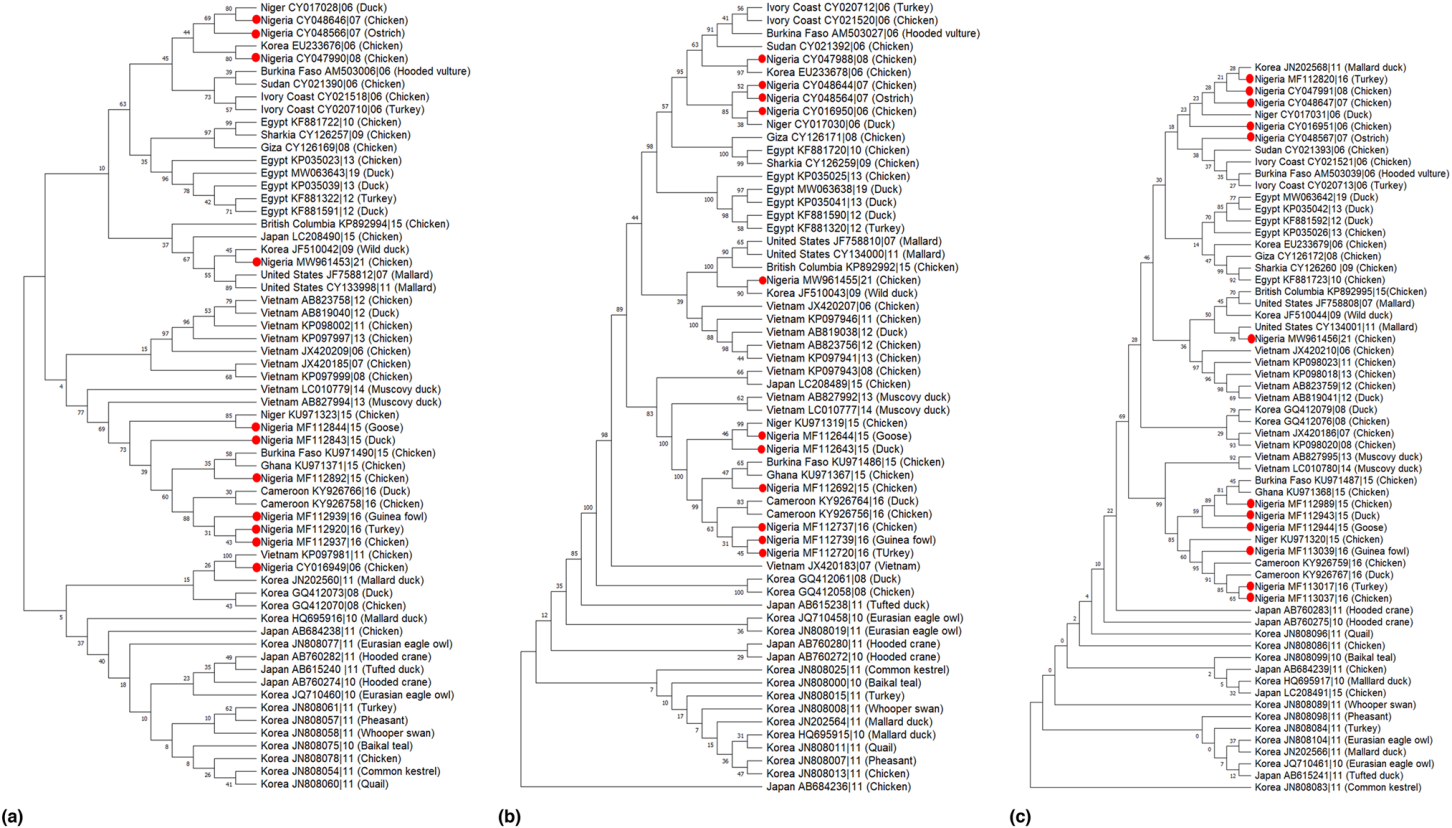
Phylogenetic relationship of H5N1 viruses from Nigeria (red dots) and other countries generated in MEGA11 (maximum likelihood analysis and 1000 bootstrap replicates) for MP (**a**), NP gene (**b**), and NS (**c**)

The phylogenetic tree for the NA gene (Fig. 3b) revealed that sequences from various years in Nigeria clustered with other sequences from African countries, except the 21 sequences isolated in 2021 that clustered with Korea and the United States. The evolutionary relationship of MP, NP, and NS gene segments in Nigeria is shown in Fig. 4. For the MP segment, 9 out of the 11 sequences in Nigeria were closely related to other African countries, while the sequences isolated in 2021 and 2006 were closely related to sequences from Korea, the United States, and Vietnam (Fig. 4a). The NP genes of the H5N1 virus isolated in Nigeria were also clustered with other African countries, except for the sequence in 2021 that was closely related to the sequence from Korea (Fig. 4b). For the NP gene segment (Fig. 4c), sequences in Nigeria were closely associated with sequences from other African countries, while sequences isolated in 2016 and 2021 were closely related to sequences from Korea and the United States, respectively.

**Fig. 5:**
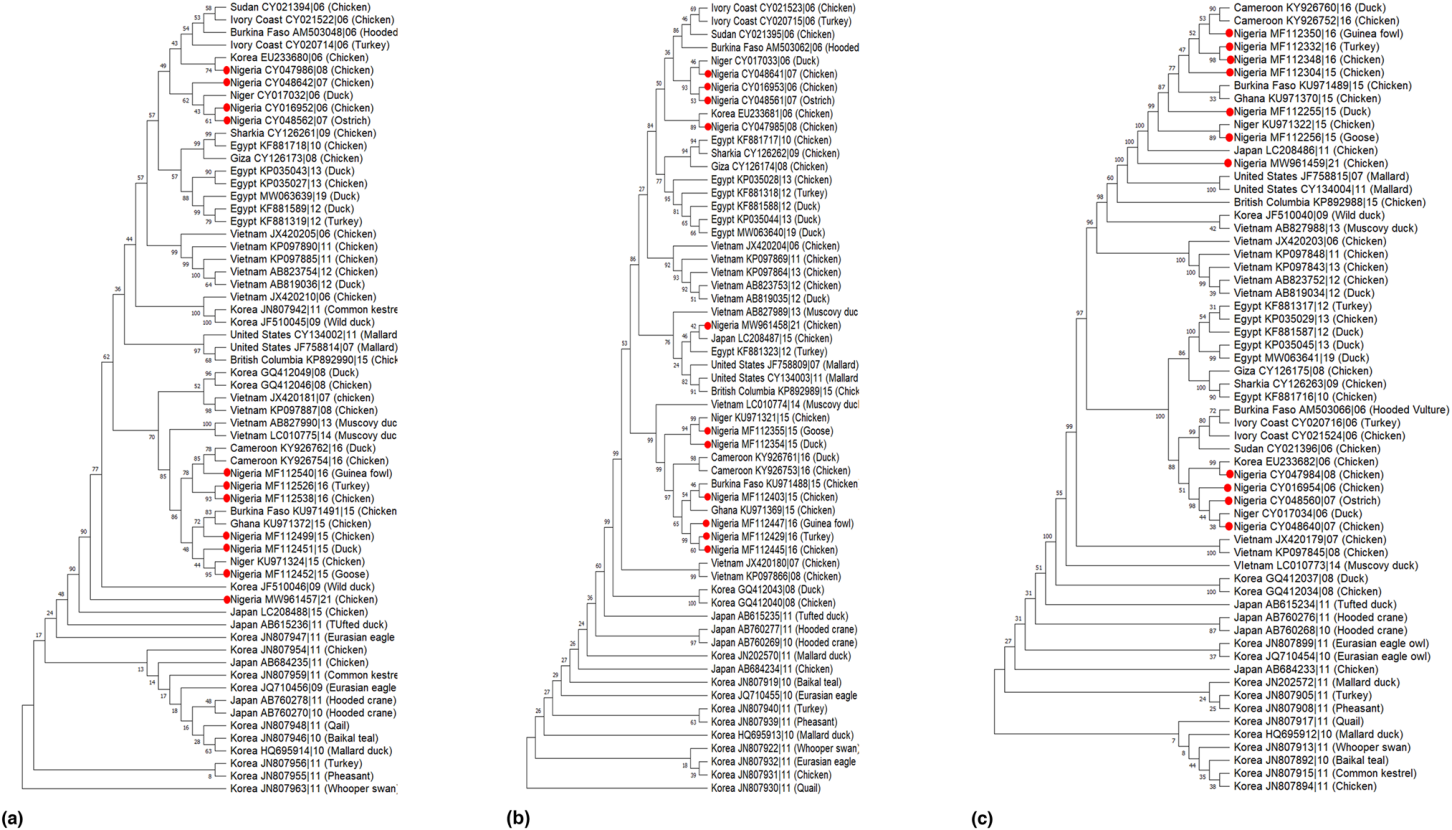
Phylogenetic relationship of H5N1 viruses from Nigeria (red dots) and other countries generated with MEGA11 (maximum likelihood analysis and 1000 bootstrap replicates) for PA (**a**), PB1 gene (**b**), and PB2 (**c**)

The evolutionary histories of PA, PB1, and PB2 genes are depicted in Fig. 5. The genetic sequences obtained from Nigeria in the PA gene segment tree showed close similarity to sequences from other African countries, except for one sequence from 2008, which was closely related to a sequence from Korea (Fig. 5a) and the sequence in 2021, which formed a separate clade from the others in Japan, Korea, and other African countries. The topology of the PB1 tree (Fig. 5b) was similar to that of the PA tree, with the sequence isolated from Nigeria in 2021 being closely related to sequences in Japan. In addition, for the PB2 tree (Fig. 5b), all the sequences obtained in Nigeria from various hosts such as guinea fowl, turkey, chicken, duck, and goose had closer ancestry sequences from other African countries.

### Clade classification

Table 1 shows the clade classification of all the isolated avian influenza sequences from 2006 to 2021. H5N1 Clade 2.2 was observed in 2006, with 2.3.2, 2.3.2.1f clades observed afterwards and 2.3.4.4b in 2021.

**Table 1:** Avian influenza H5N1 subspecies classification report

SN	Query identifier	Clade classification	Tree link
1	A/chicken/Nigeria/08VIR4337-344/2008	2.2	VIEW TREE
2	A/turkey/Nigeria/16VIR5840-79/2016	2.3.2.1f	VIEW TREE
3	A/ostrich/Nigeria/08RS848-84/2007	2.2	VIEW TREE
4	A/chicken/Nigeria/1047–34/2006	2.2	VIEW TREE
5	A/goose/Nigeria/16VIR5840-3/2015	2.3.2.1f	VIEW TREE
6	A/chicken/Nigeria/16VIR5840-51/2015	2.3.2.1f	VIEW TREE
7	A/chicken/Nigeria/VRD21-102_21VIR2370-424/2021	2.3.4.4b	VIEW TREE
8	A/duck/Nigeria/16VIR5840-2/2015	2.3.2.1f	VIEW TREE
9	A/guinea_fowl/Nigeria/16VIR5840-98/2016	2.3.2.1f	VIEW TREE
10	A/chicken/Nigeria/08RS848-99/2007	2.2	VIEW TREE
11	A/chicken/Nigeria/16VIR5840-96/2016	2.3.2.1f	VIEW TREE

## Discussion

In this study, we present valuable insight into the genetic diversity and evolutionary dynamics of AIV in Nigeria. We found several clades to be circulating in various birds, including 2.3.4.4b in 2021. The outbreaks were across 32 states, with Kano, Kaduna, and Plateau states in the north accounting for 60% of the cases [23]. The southern part of the country is more conducive for raising birds because of the harsh climate in the North. Therefore, many commercial exotic live birds are transported from the South to the North for poultry farming. An epidemiological link exists between chicken trade and AIV outbreaks in the regions [24]. The LBMs and free-range poultry are sources of AIV strains [25, 26]. Between 2006 and 2008, several cases of HPAI H5N1 were detected [27]. A wild bird was reported with LPAI H5N2 in 2008 [16]. Active surveillance resulted in the identification of the virus in a duck at an LBM [28]. These LBMs are places of AIV dissemination in Nigeria, while wetlands are points of transmission due to interactions with other avian species and humans. Nigeria will continue to experience an increased burden of avian influenza due to three major wild bird migratory routes that transverse the country from Asia and Europe, coinciding with the yearly peak periods of AIV outbreaks [29]. Secondly, the presence of migratory bird sanctuaries, especially in northern Nigeria [30], serves as a point for the introduction of novel strains of AIV, as observed during the 2015–2016 epizootics. Overall, Nigeria will still be a hotspot for AIV epizootics, which will subsequently spread to other regions of Africa, and this has been revealed by the clades that are circulating in other African countries.

Out of the sequences retrieved from OpenFlu database, H5N1 accounted for 97%, while the others were H2H5 (1%), H5N6 (0.3%), and H5N8 (2%). To determine the Phylogenetic relationship of H5N1 strains, complete genomes from 2006 to 2021 isolated from chicken, duck, goose, turkey, and guinea fowl were used. Few Nigerian sequences were used compared to sequences from Asia, the USA, Canada and Africa, providing a robust evolutionary diversity with diverse influenza virus populations. Trees were drawn based on eight gene segments to assess any immunological pressure along the genes, thereby providing information on the extent of H5N1 epizootic in this study from its emergence in 2006 until 2021. The OpenFlu and GenBank databases had limited molecular data from Nigeria. Although intermittent outbreaks were reported during this period [30], few sequences were deposited from Nigeria. The data obtained from OpenFlu for the phylogenetic analysis of the H5N1 subtype showed more cases reported in Africa after the 2006 outbreak in Nigeria. This supports the ancestry of the H5N1 [31] as in previous cases in Russia in 2005, which were assumed to be progenitors of the H5N1 strains that later spread to Europe and parts of Africa [32]. After the initial report in Nigeria, outbreaks were reported in other African countries such as Burkina Faso, Egypt, and Niger. All H5N1 strains obtained during the outbreak showed a close relationship and ancestry compared to those from previous outbreaks in Africa with distant topology.

These strains continue to spread in many geographic regions of Nigeria with no geographical confinement. This suggests that these strains transcend regional and international boundaries from East to West Africa, largely due to the activities of wild waterbirds, demonstrating the complexity of the epidemiological dynamics of AIV in Africa and beyond. Epidemiological data in Nigeria is limited; hence, we cannot extensively monitor the trajectory and evolution before 2006 due to inadequate surveillance programs, centralised diagnostic facilities, and the inability to fully characterise the specific influenza virus. It was also reported that AIVs were imported individually from Central Russia to Africa, similar to the same period in Europe [31]. This is because initial H5N1 reports in Africa had similar phylogenies with those detected in migratory wild birds from Eurasia [32]. As the H5N1 was first introduced in Nigeria, viral populations appeared to have independently evolved with mutations and several clades over the years. There is large-scale poultry production or industrial poultry sectors in Europe and parts of the world compared to limited poultry trade among African countries, hence little virus transmission across geographical spaces and susceptible host species compared to more domestic poultry trading than import or export [33].

The limitations of the study include the absence of recent data, which hindered deeper insight into the AIV molecular epidemiology and the trend of outbreaks. The study used data from 2006 to 2017, with a gap from 2018 to 2020. Also, avian influenza virus sequences are scarce on the OpenFlu database from Nigeria. With just 11 sequences, the exhaustive analysis of the genetic diversity and evolution in the region is limited. The study underscores the need for a comprehensive molecular epidemiology of AIV in Nigeria and, indeed, Africa.

## Conclusions

Transmission of AIV H5N1 is ongoing following its establishment in Nigeria. Several clades of H5N1 have been reported, with 2.3.4.4b clades reported in 2021. Since 2006, multiple outbreaks have been reported in all the states of Nigeria, resulting in the huge annual loss of millions of birds. Although little is done in Nigeria, few sequences were obtained from Ostrich, Chicken, duck, goose and guinea fowl. These findings provide insight into the evolutionary history and its potential for cross-border transmission. The study highlights the importance of continued surveillance and monitoring of AIV in Nigeria as an early warning system for future outbreaks.

## Supplementary Information

Below is the link to the electronic supplementary material.

Supplementary file1 (DOCX 26 KB)

## Data Availability

All the nucleotide sequences used in this study are publicly available on OpenFlu database (https://openflu.vital-it.ch/browse.php#results) by Swiss Institute of Bioinformatics and National Center for Biotechnology Information (https://www.ncbi.nlm.nih.gov/nucleotide/).
