# Supplementary material for: Molecular epidemiology and genetic evolution of avian influenza H5N1 subtype in Nigeria, 2006 to 2021
Source: Virus Genes. 2024 Jun 19;60(5):501–9. doi: 10.1007/s11262-024-02080-9 (PMC11383836; doi:10.1007/s11262-024-02080-9)
Supplement: Supplementary file 1 — Supplementary file1 (DOCX 26 KB) [file 11262_2024_2080_MOESM1_ESM.docx]

SUPPLEMENTARY MATERIALS

**Table S1.** Molecular evidence of AIV from Nigeria

| **S/N** | **Subtypes** | **Number of sequences** | **Percentage** |
| --- | --- | --- | --- |
| 1 | H5N1 | 280 | 97% |
| 2 | H5N2 | 3 | 1% |
| 3 | H5N6 | 1 | 0.30% |
| 4 | H5N8 | 5 | 2% |

Table S2. Description of H5N1 isolates by country, 2006 to 2021

| **Sequence ID** | **Sample collection date** | **Host** | **Countries** | **Genebank ID** | | | | | | | |
| --- | --- | --- | --- | --- | --- | --- | --- | --- | --- | --- | --- |
|  |  |  |  | **HA** | **NA** | **MP** | **NP** | **NS** | **PA** | **PB1** | **PB2** |
| A/chicken/Nigeria/1047-34/2006 | 2006 | Chicken | Nigeria | CY016947 | CY016949 | CY016948 | CY016950 | CY016951 | CY016952 | CY016953 | CY016954 |
| A/chicken/Nigeria/08RS848-99/2007 | 2007 | Chicken | Nigeria | CY048643 | CY048645 | CY048646 | CY048644 | CY048647 | CY048642 | CY048641 | CY048640 |
| A/chicken/Nigeria/08VIR4337-344/2008 | 2008 | Chicken | Nigeria | CY047987 | CY047989 | CY047990 | CY047988 | CY047991 | CY047986 | CY047985 | CY047984 |
| A/chicken/Nigeria/VRD21-102_21VIR2370-424/2021 | 2021 | Chicken | Nigeria | MW961452 | MW961454 | MW961453 | MW961455 | MW961456 | MW961457 | MW961458 | MW961459 |
| A/chicken/Nigeria/16VIR5840-51/2015 | 2015 | Chicken | Nigeria | MF112592 | MF112792 | MF112892 | MF112692 | MF112989 | MF112499 | MF112403 | MF112304 |
| A/chicken/Nigeria/16VIR5840-96/2016 | 2016 | Chicken | Nigeria | MF112637 | MF112837 | MF112937 | MF112737 | MF113037 | MF112538 | MF112445 | MF112348 |
| A/duck/Nigeria/16VIR5840-2/2015 | 2015 | Duck | Nigeria | MF112543 | MF112743 | MF112843 | MF112643 | MF112943 | MF112451 | MF112354 | MF112255 |
| A/goose/Nigeria/16VIR5840-3/2015 | 2015 | Goose | Nigeria | MF112544 | MF112744 | MF112844 | MF112644 | MF112944 | MF112452 | MF112355 | MF112256 |
| A/turkey/Nigeria/16VIR5840-79/2016 | 2016 | Turkey | Nigeria | MF112620 | MF112820 | MF112920 | MF112720 | MF113017 | MF112526 | MF112429 | MF112332 |
| A/guinea fowl/Nigeria/16VIR5840-98/2016 | 2016 | Guinea Fowl | Nigeria | MF112639 | MF112839 | MF112939 | MF112739 | MF113039 | MF112540 | MF112447 | MF112350 |
| A/ostrich/Nigeria/08RS848-84/2007 | 2007 | Ostrich | Nigeria | CY048563 | CY048565 | CY048566 | CY048564 | CY048567 | CY048562 | CY048561 | CY048560 |
| A/duck/Egypt/14VIR784-6-1328S/2013 | 2013 | Duck | Egypt | KP035038 | KP035040 | KP035039 | KP035041 | KP035042 | KP035043 | KP035044 | KP035045 |
| A/chicken/Egypt/14VIR784-5-1318S/2013 | 2013 | Chicken | Egypt | KP035022 | KP035024 | KP035023 | KP035025 | KP035026 | KP035027 | KP035028 | KP035029 |
| A/chicken/Egypt/Q2247B/2010 | 2010 | Chicken | Egypt | KF881719 | KF881721 | KF881722 | KF881720 | KF881723 | KF881718 | KF881717 | KF881716 |
| A/turkey/Egypt/S6405A/2012 | 2012 | Turkey | Egypt | KF258178 | KF881321 | KF881322 | KF881320 | KF881323 | KF881319 | KF881318 | KF881317 |
| A/chicken/Giza/CAI15/2008 | 2008 | Chicken | Egypt | CY126168 | CY126170 | CY126169 | CY126171 | CY126172 | CY126173 | CY126174 | CY126175 |
| A/chicken/Sharkia/CAI38/2009 | 2009 | Chicken | Egypt | CY126256 | CY126258 | CY126257 | CY126259 | CY126260 | CY126261 | CY126262 | CY126263 |
| A/Duck/Egypt/S75-P10-du/2019 | 2019 | Duck | Egypt | MW063636 | MW063637 | MW063643 | MW063638 | MW063642 | MW063639 | MW063640 | MW063641 |
| A/duck/Egypt/Q4596D/2012 | 2012 | Duck | Egypt | JX912994 | JX912993 | KF881591 | KF881590 | KF881592 | KF881589 | KF881588 | KF881587 |
| A/chicken/Ivory Coast/1787-35/2006 | 2006 | Chicken | Cote d'Ivoire | CY021517 | CY021519 | CY021518 | CY021520 | CY021521 | CY021522 | CY021523 | CY021524 |
| A/chicken/Sudan/2115-10/2006 | 2006 | Chicken | Sudan | CY021389 | CY021390 | CY021391 | CY021392 | CY021393 | CY021394 | CY021395 | CY021396 |
| A/turkey/Ivory Coast/4372-4/2006 | 2006 | Turkey | Cote d'Ivoire | CY020709 | CY020711 | CY020710 | CY020712 | CY020713 | CY020714 | CY020715 | CY020716 |
| A/chicken/Niger/15VIR2060-14/2015 | 2015 | Chicken | Niger | KU971317 | KU971318 | KU971323 | KU971319 | KU971320 | KU971324 | KU971321 | KU971322 |
| A/duck/Niger/914/2006 | 2006 | Duck | Niger | CY017027 | CY017029 | CY017028 | CY017030 | CY017031 | CY017032 | CY017033 | CY017034 |
| A/Hooded vulture/Burkina Faso/2/2006 | 2006 | Hooded Vulture | Burkina Faso | AM400972 | AM503017 | AM503006 | AM503027 | AM503039 | AM503048 | AM503062 | AM503066 |
| A/chicken/Burkina Faso/15VIR1774-2/2015 | 2015 | Chicken | Burkina Faso | KU971484 | KU971485 | KU971490 | KU971486 | KU971487 | KU971491 | KU971488 | KU971489 |
| A/chicken/Cameroon/16VIR3791-16/2016 | 2016 | Chicken | Cameroon | KY926755 | KY926757 | KY926758 | KY926756 | KY926759 | KY926754 | KY926753 | KY926752 |
| A/duck/Cameroon/16VIR3791-21/2016 | 2016 | Duck | Cameroon | KY926763 | KY926765 | KY926766 | KY926764 | KY926767 | KY926762 | KY926761 | KY926760 |
| A/chicken/Ghana/15VIR2588-11/2015 | 2015 | Chicken | Ghana | KU971365 | KU971366 | KU971371 | KU971367 | KU971368 | KU971372 | KU971369 | KU971370 |
| A/chicken/Korea/IS/2006 | 2006 | Chicken | South Korea | EU233675 | EU233677 | EU233676 | EU233678 | EU233679 | EU233680 | EU233681 | EU233682 |
| A/chicken/Korea/ISQ250/2008 | 2008 | Chicken | South Korea | GQ412051 | GQ412063 | GQ412070 | GQ412058 | GQ412076 | GQ412046 | GQ412040 | GQ412034 |
| A/duck/Korea/JEQ149/2008 | 2008 | Duck | South Korea | GQ412052 | GQ412067 | GQ412073 | GQ412061 | GQ412079 | GQ412049 | GQ412043 | GQ412037 |
| A/Mallard duck/Korea/W401/2011 | 2011 | Mallard Duck | South Korea | JN202558 | JN202562 | JN202560 | JN202564 | JN202566 | JN202568 | JN202570 | JN202572 |
| A/mallard/Korea/1195/2010 | 2010 | Mallard Duck | South Korea | HQ695910 | HQ695911 | HQ695916 | HQ695915 | HQ695917 | HQ695914 | HQ695913 | HQ695912 |
| A/wild duck/Korea/CSM4-12/2009 | 2009 | Wild Duck | South Korea | JF510041 | JF510047 | JF510042 | JF510043 | JF510044 | JF510046 | JF510045 | JF510040 |
| A/Eurasian eagle owl/Korea/23/2010 | 2010 | Eurasian Eagle Owl | South Korea | JQ710457 | JQ710459 | JQ710460 | JQ710458 | JQ710461 | JQ710456 | JQ710455 | JQ710454 |
| A/baikal teal/Korea/Q524/2010 | 2010 | Baikal Teal | South Korea | JN807976 | JN808028 | JN808075 | JN808000 | JN808099 | JN807946 | JN807919 | JN807892 |
| A/chicken/Korea/YS171/2011 | 2011 | Chicken | South Korea | JN807982 | JN808034 | JN808078 | JN808013 | JN808086 | JN807954 | JN807931 | JN807894 |
| A/eurasian eagle owl/Korea/Q182/2011 | 2011 | Eurasian Eagle Owl | South Korea | JN807974 | JN808032 | JN808077 | JN808019 | JN808104 | JN807947 | JN807932 | JN807899 |
| A/quail/Korea/GC395/2011 | 2011 | Quail | South Korea | JN807995 | JN808030 | JN808060 | JN808011 | JN808096 | JN807948 | JN807930 | JN807917 |
| A/common kestrel/Korea/Q197/2011 | 2011 | Common Kestrel | South Korea | JN807983 | JN808036 | JN808054 | JN808025 | JN808083 | JN807959 | JN807942 | JN807915 |
| A/whooper swan/Korea/Q28/2011 | 2011 | Whooper Swan | South Korea | JN807998 | JN808041 | JN808058 | JN808008 | JN808089 | JN807963 | JN807922 | JN807913 |
| A/pheasant/Korea/PT411/2011 | 2011 | Pheasant | South Korea | JN807994 | JN808048 | JN808057 | JN808007 | JN808098 | JN807955 | JN807939 | JN807908 |
| A/turkey/Korea/DDC518/2011 | 2011 | Turkey | South Korea | JN807996 | JN808047 | JN808061 | JN808015 | JN808084 | JN807956 | JN807940 | JN807905 |
| A/muscovy duck/Vietnam/LBM330/2013 | 2013 | Muscovy Duck | Vietnam | AB827991 | AB827993 | AB827994 | AB827992 | AB827995 | AB827990 | AB827989 | AB827988 |
| A/duck/Soc Trang/8/2012 | 2012 | Duck | Vietnam | AB819037 | AB819039 | AB819040 | AB819038 | AB819041 | AB819036 | AB819035 | AB819034 |
| A/chicken/Vietnam/NCVD-A937/2011 | 2011 | Chicken | Vietnam | KP097925 | KP097981 | KP098002 | KP097946 | KP098023 | KP097890 | KP097869 | KP097848 |
| A/chicken/Vietnam/NCVD-A015/2008 | 2008 | Chicken | Vietnam | KP097922 | KP097978 | KP097999 | KP097943 | KP098020 | KP097887 | KP097866 | KP097845 |
| A/chicken/Vietnam/NCVD-KA448/2013 | 2013 | Chicken | Vietnam | KP097920 | KP097976 | KP097997 | KP097941 | KP098018 | KP097885 | KP097864 | KP097843 |
| A/muscovy duck/Long An/90/2014 | 2014 | Muscovy Duck | Vietnam | LC010776 | LC010778 | LC010779 | LC010777 | LC010780 | LC010775 | LC010774 | LC010773 |
| A/chicken/Soc Trang/2/2012 | 2012 | Chicken | Vietnam | AB823755 | AB823757 | AB823758 | AB823756 | AB823759 | AB823754 | AB823753 | AB823752 |
| A/chicken/Cao Bang/20/2007 | 2007 | Chicken | Vietnam | JX420182 | JX420184 | JX420185 | JX420183 | JX420186 | JX420181 | JX420180 | JX420179 |
| A/chicken/Ca Mau/1180/2006 | 2006 | Chicken | Vietnam | JX420206 | JX420208 | JX420209 | JX420207 | JX420210 | JX420205 | JX420204 | JX420203 |
| A/hooded crane/Kagoshima/110214-3/2011 | 2011 | Hooded Crane | Japan | AB760279 | AB760281 | AB760282 | AB760280 | AB760283 | AB760278 | AB760277 | AB760276 |
| A/hooded crane/Kagoshima/4612J004/2010 | 2010 | Hooded Crane | Japan | AB760271 | AB760273 | AB760274 | AB760272 | AB760275 | AB760270 | AB760269 | AB760268 |
| A/chicken/Chiba/2/2011 | 2011 | Chicken | Japan | AB684262 | AB684237 | AB684238 | AB684236 | AB684239 | AB684235 | AB684234 | AB684233 |
| A/tufted duck/Fukushima/2/2011 | 2011 | Tufted Duck | Japan | AB615237 | AB615239 | AB615240 | AB615238 | AB615241 | AB615236 | AB615235 | AB615234 |
| A/chicken/Japan/AQ-HE79/2015 | 2015 | Chicken | Japan | LC208484 | LC208485 | LC208490 | LC208489 | LC208491 | LC208488 | LC208487 | LC208486 |
| A/mallard/Maryland/802/2007 | 2007 | Mallard | USA | JF758813 | JF758811 | JF758812 | JF758810 | JF758808 | JF758814 | JF758809 | JF758815 |
| A/mallard/California/2536P/2011 | 2011 | Mallard | USA | CY133997 | CY133999 | CY133998 | CY134000 | CY134001 | CY134002 | CY134003 | CY134004 |
| A/chicken/BC/FAV2/2015 | 2015 | Chicken | Canada | KP892991 | KP892993 | KP892994 | KP892992 | KP892995 | KP892990 | KP892989 | KP892988 |
